# A Comprehensive Study into the Possibility of Integrating Shredded Recycled Tires as Aggregate in the Manufacture of Traditional Earth Blocks

**DOI:** 10.3390/polym18121520

**Published:** 2026-06-18

**Authors:** Carlos Alberto Casapino-Espinoza, José Manuel Gómez-Soberón, María Consolación Gómez-Soberón

**Affiliations:** 1 Barcelona School of Architecture, Polytechnic University of Catalonia, Avenida Diagonal 649, 08028 Barcelona, Spain; carlos.alberto.casapino@upc.edu; 2 Department of Architecture Technology, Barcelona School of Building Construction, Polytechnic University of Catalonia, Av. Doctor Marañon 44-50, 08028 Barcelona, Spain; 3 Civil Engineering School, Metropolitan Autonomous University, Av. San Pablo 180, Mexico City 2200, Mexico; cgomez@azc.uam.mx

**Keywords:** waste, sustainable aggregates, shredded tire, earth blocks

## Abstract

The current research evaluates the potential of incorporating shredded end-of-life tires as recycled aggregate in traditional earth blocks, proposing a sustainable alternative for the managing and valorization of this waste. Shredded tire particles at the upper granulometric limit, according to applicable regulations for this type of block, were used in various volume replacement percentages. The results reveal that the bulk density remains almost constant, increasing by 2.12% after 20% replacement, while the porosity increases progressively with reduced content, reaching a maximum of 17.63% for the same replacement. Although the mechanical properties decrease with higher replacement percentages, reaching 2.061 MPa with a 31.83% reduction in compressive strength and a 30.18% reduction in flexural strength compared to the control samples, these values still exceed regulatory requirements. In contrast, there is an optimization of thermal properties, with a minimum conductivity value of 0.66 W/m·K and improvements in erosion resistance, including reductions of up to 42.71%. Through Thermogravimetric Analysis and Optical Image Analysis tests, complementing the feasibility analysis, it is determined that this type of block is viable for masonry applications for light or non-structural loads. Likewise, the material exhibits significant improvements in erosion resistance and highlights its thermal behavior as a potential insulating element. However, polymer degradation when exposed to high temperatures limits its application due to the loss of mechanical stability and the potential risks associated with matrix degradation.

## 1. Introduction

According to the International Rubber Study Group (cited in [1]), an estimated 17.1 million tons of tires reached the ‘end of life tires’ (ELTs) phase worldwide in 2018. Meanwhile, a representative study with data from 19 countries reported that such tires amount to 25,676 kilotons, of which of 17,158 kilotons are recovered for recycling and 17,712 kilotons are involved in engineering and backfill applications [2].

Therefore, the management of ELT waste is one of the main challenges in terms of the growth of the world’s vehicle fleet, which in turn presents many different challenges. These include the overloading of landfills and the saturation of treatment systems due to the quantities and volumes produced, the availability of space for waste disposal [3], the lack of adequate treatment infrastructure, low efficiency in resource separation, a high proportion of poorly managed waste, pollution problems related to leaching and the release of toxic substances [3,4], the lack of effective regulation and economic incentives and, finally, issues connected with the intervention of the informal sector.

According to the ASTM D5603-19A standard [5], the composition is variable, consisting mostly of natural rubber (19%), synthetic polymers (24%), textiles (4%), reinforcing steel wire (12%), black carbon or silica filler (26%) and products such as antioxidants and antiozonants (14%) [6]. These elastomers consist mainly of blends of natural rubber (NR), copolymers of styrene-butadiene (SBR), and polybutadiene [7].

These compounds exhibit a 99% recycling potential [3] and are mainly used for material recovery, energy recovery or other uses in civil engineering [1,2]. Alternatives such as pyrolysis represent a viable option for energy production [8], although the gases produced generate pollution in the local atmosphere [6,9], which also poses a health risk [3].

E.M. Palmeira et al. [10] points out that these applications provide economic and eco-friendly solutions (such as in geotechnical and geoenvironmental works), where the risk of contamination in soil-rubber mixtures is low and the resistance to degradation is high [3]. Thus, the use of tire waste is viable as a sustainable strategy to divert this waste material, representing an opportunity to achieve sustainability goals. However, it is noted that further research is required regarding compositional variability, long-term effects, and contamination impacts caused by degradation [11].

Among the most common uses, retaining walls for slope stabilization [12], lightweight fills, soil reinforcement, erosion control, acoustic barriers, thermal insulation, and the integration of these by-products into construction materials to provide lightness, durability, compressibility, and thermal and acoustic insulation capacity stand out. Another common use is the valorization as a by-product through crushing (crushed tire waste—CTW) to obtain particle sizes such as in ground tire rubber (GTR), rubber mulch (RM), and fibrous rubber particles (FRPs). These are used in bituminous mixtures for rubberized asphalt [13] and binder modifiers or as sustainable aggregate [14,15]. Although they are used in artificial turf fields, a potential health risk was found due to the dermal exposure, inhalation, and ingestion of heavy metals and leached compounds [16,17], with impacts similar to conventional urban pollution.

Likewise, M. Ul Islam et al. [18] mention that the porous nature of rubber near water tables causes leaching of toxic substances and pollutants, while its disposal through heat exposure or combustion produces compounds such as benzene and toluene [19]. To mitigate this, various studies propose encapsulating or embedding CTW as a partial substitute for aggregates in cement matrices [20,21], asphalt [22], and soil [23,24]. They report that no leaching of encapsulated tires is observed [25,26]; in the case of soil matrices, clay absorbs the zinc and organic carbon leachate from rubber particles and mineral oxides [27,28]. It was also found that the tire particles themselves absorb certain metals [29]. Therefore, the incorporation of CTW in soil matrices is viable if the tire particles remain encapsulated and protected from direct water exposure.

From previous research, the incorporation of granulated tires into concrete mixtures stands out [3], through the partial substitution of fine or coarse aggregate. The size and morphological characteristics (such as the surface texture) of the GTR depend on the shredding technique and processes [30,31,32], which in turn affect their suitability for the intended application.

The literature agrees on the proportional relationship (in some cases linear or semi-linear) between the reduction in mechanical strength and the higher tire contents [33,34], with reductions of up to 200% being reported [35]. This is due to the reduction of the elasticity modulus (which exhibits greater deformation than mineral aggregates), the weakness of the interfacial transition zone (ITZ) and the increase in porosity [4]. However, at relatively low amounts (typically less than 10–15% by volume), it is possible to obtain acceptable values under applicable regulatory parameters, ideal for non-structural masonry applications. It is noted that induced porosity increases the water absorption (which can affect moisture cycle behavior) and durability [4].

Despite the mechanical strength values reported due to the incorporation of CTW particles (for a compressive strength between 2.8 and 40 MPa as typical values) [36,37], other favorable effects are reported, such as a reduction of bulk density, increases in ductility, toughness, and deformability, and improvements in thermal and acoustic performance [4,38]. The increase in ductility optimizes energy absorption against impacts or dynamic loads [39,40]. The improvement in acoustic behavior benefits from the combined mechanisms of the viscous friction and viscoelastic damping of rubber.

### Incorporating Tires into Earth-Based Masonry Materials

Interest in incorporating CTW into the manufacture of compressed earth masonry elements, bricks, blocks, adobes and mortars has increased significantly [41,42]. Several studies agree on its practical utility due to the aforementioned benefits.

As with cement matrices, porosity leads to a reduction in mechanical strength that depends on the substitution percentages and the aggregate particle size distribution. At low replacement percentages, it is possible to maintain the values of mechanical resistance within the regulatory parameters for non-structural bricks. On the other hand, higher replacement contents compromise the structural integrity of the block. The main factors influencing the behavior include aggregate size (such as fine aggregate [7,43] or coarse aggregates [35]), replacement percentage, surface treatment of the particles [44], and the matrix constitution.

Smaller aggregates are distributed more homogeneously and generate lower stress concentrations, although in the case of Fibrous rubber particles (FRPs) they contribute to improving mechanical performance [44]. Therefore, it is necessary to calculate the effect of the larger particle ratios by measuring the strength–porosity relationship to validate regulatory strength limits and the thermal benefits of increased interstitial voids.

A systematic literature review suggests that the integration of CTW into soil matrices decreases the carbon footprint associated with the cycle and the thermal capacities generated [45]. However, further microstructural studies are still required to reduce the variability of the results regarding grain or fiber size for industrial application.

From previous experimental results, evidence shows that tire waste increases water absorption, contributes to mass loss, and achieves reduced thermal conductivity (λ) values. Regarding this property, values up to 1.85 W/m·K [46] are reported in concrete matrices for moderate replacements, ranges of 0.24–0.36 W/m·K for replacements greater than 50% and with trapped air [47], and values of 0.5–2.0 W/m·K [48] for stabilized soils. It is seen that the incorporation of CTW significantly reduces λ, which improves its performance as a thermal building envelope; however, the reduction in mechanical strength and increases in porosity do not recommend it for use in structural elements such as load-bearing walls.

In terms of regulatory criteria, there is no framework that directly regulates earth blocks with ELTs, but existing standards justify the technical use of ELTs alongside those concerning soil use and earth block manufacturing, such as ASTM D6270-17 [49] (without lime, cement, or other additives, which fall outside the scope of this research); aggregate replacement criteria must be carefully managed to correctly interpret the results, using standards such as IS 1077 [50], UNE 41410 [51], E-080 [52], and ASTM C62 [53], or those relevant nationally) as reference frameworks.

Regarding leaching risk, although clay has been shown to retain certain compounds, recent regulations such as European Union (EU) regulation 2023–2055 [54], ISO 24187:2023 [55], and ASTM D8489-23e1 [56] restrict the release of microplastics into the environment and address toxicity aspects. Nonetheless, no specific studies were found on the long-term degradation of earth matrices with CTW for masonry, meaning further studies and new lines of research are necessary; however, due to its breadth, it falls outside the initial objectives and is not addressed in this article.

Finally, few studies exist on the granular use of ELTs with dimensions exceeding those considered small but limited by the maximum (large) threshold, and moisture evaluations and curing effect studies are also limited; these factors may contribute to improving thermal performance. The results demonstrating that density reduction and increased porosity rule out these blocks due to adverse effects on mechanical properties make it necessary to determinate the appropriate balance between mechanical and thermal performance. Furthermore, stabilization or compaction reduces the porosity that the tire beneficially generates; therefore, it is necessary to determine the behavior of unstabilized blocks according to traditional, sustainable, and low-embodied-energy manufacturing criteria [11].

Accordingly, the present study aimed to investigate the physical, mechanical and thermal properties of uncompacted earth blocks incorporating recycled crushed tire aggregate with dimensions falling within the upper regulatory limit (due to the insulation and pore-generation advantages), across different dosages, given the various optimization possibilities attributable to aggregate replacement in this type of matrix.

## 2. Materials

**Figure 1:**
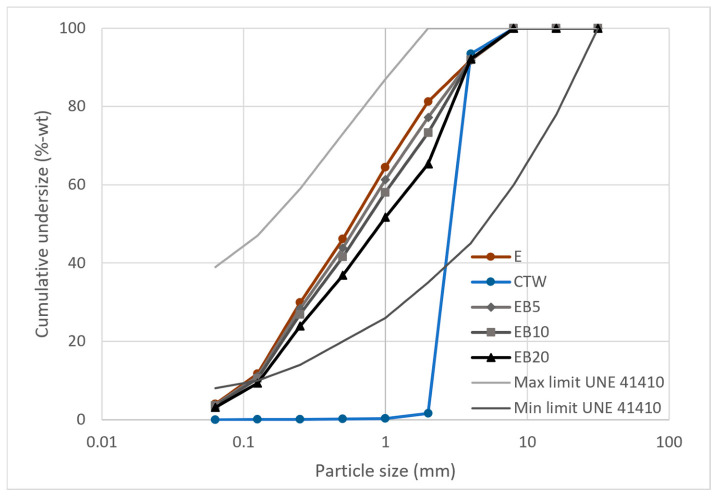
Granulometry of CTW and the different replacement percentages of study (The associated replacement percentages are explained in the Dosage Section). Note: The particle size curve of E (Extracted from [61] and the particle size curve of CTW are also presented, with the different replacement percentages in accordance with UNE-EN 14243-2:2019 [57].

For this research, the crushed tire waste (CTW) of ELTs was studied as a replacement aggregate in clay soil with water for the manufacture of uncompacted and unstabilized EBs. The CTW was obtained from a waste management company near the city of Lleida, Spain (41°21′57” N 0°30′44” E), having been previously treated and crushed. This process allows its use as a second-generation aggregate. The particle size used (in accordance with UNE-EN 14243-2 [57] and UNE 53936:2015 EX [58]) is presented in Figure 1, as determined in accordance with ASTM E11/95 [59] and UNE 7050-2 [60]. The parameter of the E-080 [52] standard was considered for the maximum particle size (as a criterion for the use of aggregates in the manufacture of EBs). The chemical characterization of the aggregate particles was performed by thermogravimetric analysis (TGA) and is described in Section 4.8.

The need to set up a controlled void structure led to a replacement aggregate with a higher coarse content being adopted (up to 5 mm according to E-0.80). Limiting the amount of fine aggregate that acts as filling material prevents the clogging of natural interstices and promotes an increase in the porosity index. Similarly, the replacement aggregate’s specific surface is reduced by using this maximum nominal size, which improves matrix encapsulation and strengthens potential deterioration against external agents.

The earth (E) used in this study showed its suitability for use in the EB factory. This has been studied, described and validated in previous research [61]. The water used for the characterization and constitution of the matrices is considered potable, complying with the requirements of the E-080 [52] standard for the manufacture of this type of block; it also has certification from Aigües de Barcelona‘s drinking water quality reports in conformity with the RD 3/2023 standard [62].

The CTW aggregate has a specific gravity of 526.12 (kg/m^3^) according to ASTM C128-22 [63]. The granulometric analysis (Figure 1) was carried out according to the UNE-EN ISO 17892-4:2019 [64] standard, in which the coarse particle content is notable. UNE 41410 [51] and ASTM C144-18 [65], which describe a fineness modulus (FM) of 6.04, are taken as references for maximum and minimum particle size restrictions.

According to the granulometric analysis data, 0.278% of the material passed through sieve No. 18 (1.00 mm). Therefore, the UNE 53936:2015 EX [58] standard is not applicable for determining the content of textile fibers, as ELT powder of a nominal size less than 0.8 mm is excluded from the analysis. Similarly, the UNE-EN 14243-2 [57] standard is not considered applicable, since the CTW has already been processed and is considered treated and free of ferromagnetic impurities.

The moisture content (MC) of the CTW was determined according to UNE-EN ISO 17892-1:2015 [66] and DIN EN 16916:2025-02 [67] (at 100 °C for 24 h) for samples stored indoors at 24 °C and 60% relative humidity (RH) and samples stored outdoors. Contents of 1.26 and 1.51% were obtained, respectively, being attributable to surface and capillary free water; thus, the CTW is considered a non-absorbent material with minimal surface water retention. The MC of E obtained under the same procedure equals 3.69% and is taken from [61]. To determine the replacement percentages for CTW aggregate, the NZS 4298 [68] and UNE 41410:2023 [51] standards were used as a reference. These standards establish guidelines for earth blocks (EBs) and stabilized soil blocks (SSBs), including particle size distribution and fineness modulus criteria that ensure adequate particle size distribution. Previous studies [24,69,70] related to the physical-mechanical behavior of similar matrices were also analyzed. In these studies, substitutions between 30% and 40% showed significant losses in mechanical performance and cohesion as well as increases in porosity, among other effects. In parallel, the characteristics of the soil aggregate obtained during its characterization were considered, as well as the properties of the constituent matrices, including mechanical behavior, thermal properties, and porosity. Based on this, a maximum substitution percentage of 20% by volume was defined, considered the theoretical optimum in terms of balance between the evaluated properties.

Furthermore, it was observed that variations in the properties begin to appear with substitution percentages between 2% and 6%, which is why a minimum value of 5% was established. Additionally, an intermediate level of 10% was incorporated to analyze the evolution of the properties starting from a medium substitution percentage. Therefore, for this study, CTW was considered as an aggregate, using substitution percentages of 5%, 10%, and 20% by volume. These are compared to a control matrix (named EBXX, where XX is the proportion of aggregate replacement), which did not have aggregate replacement for any of the tests.

The liquid limit (LL) of E described In [61] is considered with regard to the water content in the mixtures of E with CTW, with the proportions of MC as a function of the replacement percentages of CTW resulting in 17.89, 17.00, 16.10 and 14.31% of MC for EB0, EB5, EB10 and EB20, respectively.

A prior study [45] used a comprehensive review of the literature to discuss the advantages, in terms of their mechanical and thermal properties, of incorporating shredded and granulated tires into compacted earth blocks. It was decided to study the incorporation of CTW in uncompacted or stabilized matrices to take advantage of the porosity and resulting density in earth matrices, in addition to using the traditional manufacturing process described in [61].

## 3. Methods

Once the materials were characterized and the dosages determined, the samples were manufactured following the specifications of standard E-080 [52]. The process was the same as that developed and detailed in previous research [61], which consisted of mixing the materials in a Guy Noel B150 (Altrad, Florensac, France) electric concrete mixer, 150 L capacity, 25 rpm (after saturating the internal walls to prevent moisture loss). Once mixed, the mixture was transferred to 50 L containers to rest for 24 h to activate the clay and distribute moisture. Then, it was poured in approximately 2 cm layers into wooden molds (pine with layers of water-repellent product), which had been moistened before pouring. Each mold was constructed with the dimensions of the specimens for subsequent tests.

Drying and curing were carried out for 90 days under protected conditions to ensure a gradual loss of moisture, reaching a maximum of 2%, which was verified using a Kern digital scale, model KB 3600-2N (Balingen, Germany) (3600 g capacity and 0.01 g precision), and a Gram HGM Series digital scale (Horsens, Denmark) (20 kg capacity and 1 g precision).

To avoid boundary conditions resulting from manufacturing, the specimens with nominal dimensions of 5 × 5 × 10, 5 × 5 × 20, and 10 × 10 × 10 were polished to ensure accurate data collection and the proper execution of subsequent tests.

An experimental campaign, consisting of several tests to assess various properties, was conducted in order to investigate the impacts resulting from the addition of CTW aggregate to the earth matrices.

**Figure 2:**
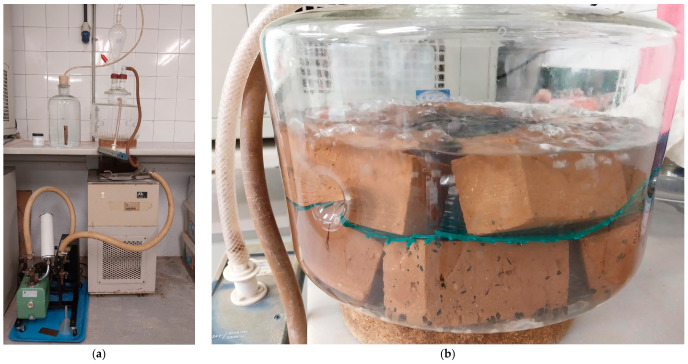
Porosity and density test by immersion. (**a**) Vacuum equipment. (**b**) Matrices during test execution.

The bulk density, bulk solid density and porosity were determined according to UNE-EN ISO 18754:2022 [71], with immersion in alcohol and application to four specimens with nominal dimensions of 5 × 5 × 10 cm. The equipment used was a Telstar SA-Torricelli Mod. CD-6 (Terrassa, Barcelona, Spain), equipped with a cold trap to prevent moisture from entering the motor. Once the vacuum was established (duration 15 min), the specimens were completely immersed in alcohol. The specimen container was then adjusted to atmospheric pressure, and the saturated and submerged weights were determined. The dry weight was determined by drying the samples in an oven for five days at 60 ± 5 °C to prevent disintegration due to specimen shrinkage or possible volatilization of tire compounds. Using the above parameters, the standard formulations were applied to obtain the solid bulk density, bulk density, and pore content (Figure 2), considering the maximum, minimum, and average results and the standard deviation.

**Figure 3:**
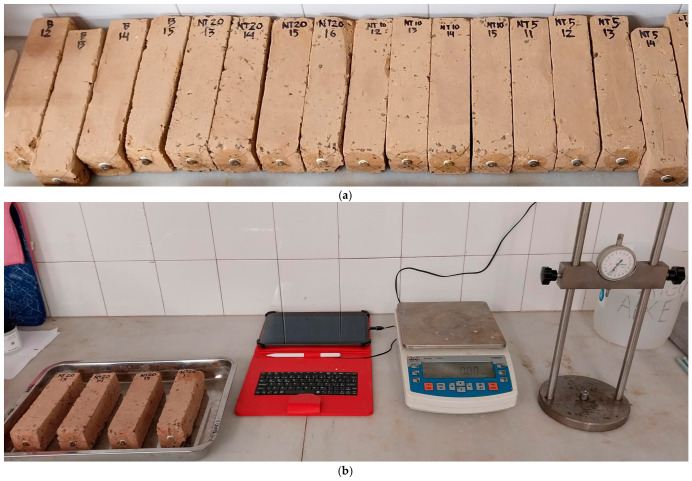
(**a**) Test specimens used for expansion testing. (**b**) Equipment used for measurement after exposure to humidity and drying processes.

The shrinkage and shrinkage limit in the new matrices could not be determined according to the AS 1289.3.4.1-2008 [72] test, due to the hydrophobicity and particle size of the CTW RA. Therefore, it was decided to perform the test using the hygroscopic expansion test method, according to the methodology detailed in a previous study [61], where the procedures and principles of ASTM C426-22 [73] and UNE-EN 1367-4 [74] were taken as a reference. This test was carried out on four specimens with nominal dimensions of 5 × 5 × 20 cm (Figure 3), considering all the results and obtaining the average of each variable. In each test specimen, two hydraulic shrinkage anchor pins were placed on the faces using a hydrophobic and heat-resistant adhesive (Ceys-Montack, Barcelona, Spain). These reference points were used to determine the dimensional changes, which were measured with a Mitutoyo precision micrometer (Kawasaki, Japan) with an accuracy of 0.0001 mm.

The process included the following steps: The samples were oven-dried for 72 h at 60 ± 5 °C, and then their temperature was stabilized by placing them in an acrylic climate chamber (equipped with silica gel) for 24 h at 20% relative humidity (RH). Subsequently, they were left for another 24 h at 20 °C and 45% RH, at which point the first determination of dimensional change was performed. All subsequent determinations were performed using samples placed in a humid chamber (20 °C and 100% RH) every 24 h for seven days. Weight was established using a Kern balance with a maximum capacity of 3600 g and a precision of 0.01 g, to determine the moisture content of the samples along with the volumetric changes.

**Figure 4:**
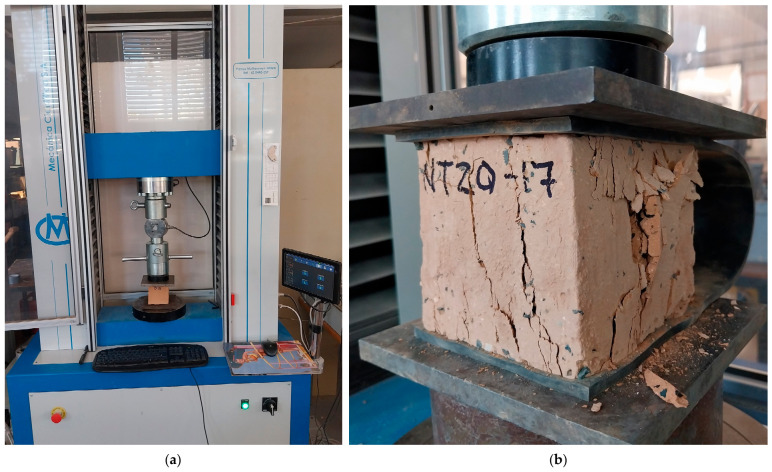
(**a**) Test equipment and configuration. (**b**) Fractured specimen after ultimate strength testing.

To determine the compressive strength (*fc*), the following standards were adapted together: ASTM C39 [75], from which the criteria related to the axial and continuous application of the compressive load, the use of rigid metal plates to ensure a uniform distribution of stresses, and the calculation of compressive strength by means of the relationship between the maximum load and the loaded area were adopted; ASTM C-67 [76] from which the criteria associated with conditioning the support surfaces using elastomeric elements were taken, employing a neoprene layer on each contact face to reduce surface irregularities and avoid local stress concentrations during the test; UNE-EN 772-1:2011+A1:2016 [77], from which the use of a minimum set of six test specimens was adopted, as was the criterion of obtaining the representative average strength of the material from the average of the individual results; and E-080 [52], which provided guidelines for applying the test to soil materials, particularly regarding the use of prismatic or cubic specimens, and for the mechanical evaluation of soil-based construction units, standards that were adapted together. A 5.4 mm neoprene sheet and a 9 mm metal plate were used for the test in order to guarantee the distribution of the load applied to the contact surfaces on both sides of the specimen. Six samples with nominal dimensions of 10 × 10 × 10 cm were tested, considering all the results and obtaining the average. The equipment used was a multi-test press of the Mecánica Científica SA brand (Getafe, Spain), with a capacity of 300 KN. Although standards such as ASTM C39 establish higher loading rates for high-strength cementitious materials, a load speed of 3 N/min was applied as an experimental adaptation for earth materials, considering that these types of materials exhibit lower resistance values and greater sensitivity to rapid loading compared to conventional materials such as concrete. Reducing the loading rate ensured a gradual transfer of stresses, minimized inertial effects, and allowed for monitoring the cracking and progressive collapse of the specimens, thus obtaining a more representative record of the material’s mechanical behavior (Figure 4).

The flexural strength (*fb*) test was performed according to the three-point test in ASTM C-67 [76], which was used to adopt the criteria related to the mechanical evaluation of individual masonry units, the recording of the maximum breaking load, and the obtaining of representative values through the testing of multiple specimens. Likewise, the criterion of using a set of six samples per mix design was adopted from this standard to ensure statistical representativeness and minimize the influence of material variations. ASTM C1161-13 [78] was used for the guidelines corresponding to the mechanical configuration of the three-point bending test, using two lower supports separated by a distance of 30 cm and a point load applied vertically at the center of the span. Similarly, the criteria associated with the correct alignment of the loading system, the uniform distribution of stresses, and the monotonic and controlled application of the load during the test were adopted. A total of six standard specimens of nominal dimensions 10 × 20 × 40 cm were used, considering all the results and obtaining the average. The equipment used was the same as in the compressive strength test, except that the speed applied was 0.25 N/min in order to allow the properties to be obtained correctly with the following configuration: two metal cylinders (Φ = 40 mm) as lower support points, at an axial distance of 30 cm and centered on the specimen; in the upper part, another support point was also centered on the specimen.

**Figure 5:**
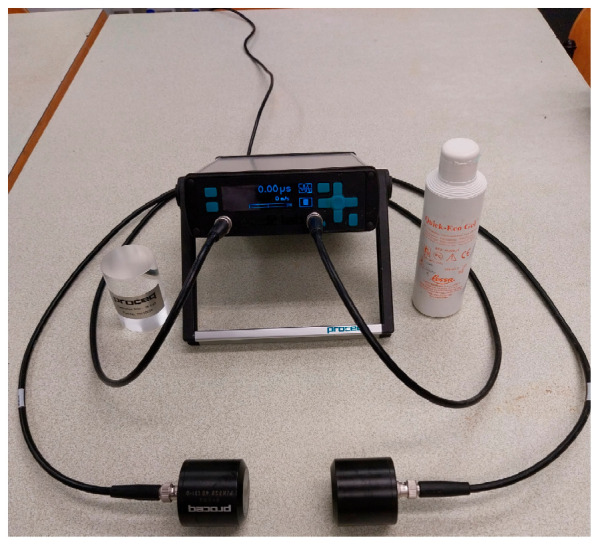
Equipment used for Ultrasonic Pulse Velocity by Wave Transmission (UPV) test.

The elastic modulus (Young) was determined by the Ultrasonic Pulse Velocity by Wave Transmission (UPV) test, in accordance with ASTM e 1876-22 [79] and UNE-EN 14146:2004 [80]. Four specimens measuring 5 × 5 × 20 cm were used, and the mean of each variable was recorded. The test followed the guidelines of the previous study [61]. The instrument used was a Proceq brand Pundi Lab (+) model (Proceq SA, Schwerzenbach, Switzerland) (Figure 5).

**Figure 6:**
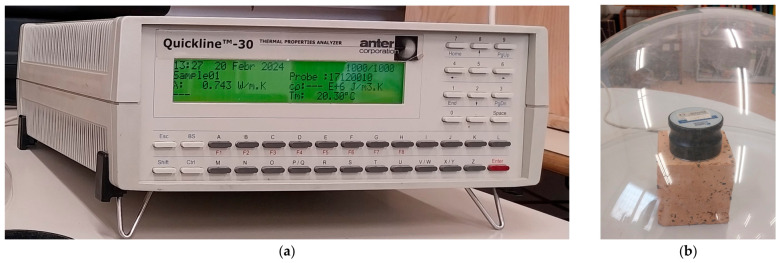
(**a**) Equipment used for the thermal property analysis test. (**b**) Thermal probe and bell configuration for thermal property analysis of test specimens.

According to previous studies [45,81] CTW shows good thermal behavior, so its use in stabilized earth blocks (SEBs) would be subject to the provisions of UNE 41410 [51], which states that information must be provided on the thermal properties of blocks for use in buildings that are subject to thermal insulation requirements. The modified transient pulse method [82] (ASTM D5334 [83] and ASTM D5930 [84]) was used. The specimens were conditioned for 24 h in a climate chamber at 20 °C with a relative humidity (RH) between 20 and 40% before the test using a QuicklineTM-30 thermal property analyzer (West Sussex, UK) (Figure 6a) with a CCI climatic chamber and an Applied Precision probe with a thermal capture range of 0.3 to 2.0 W/m·K. This equipment allows the properties of λ, diffusivity and specific heat to be obtained, based on the transient principle of the hot wire, by detecting the temperature on the contact surface with the probe. The configuration and parameters of the test correspond to those in [85] (Figure 6b). The test was performed by placing each sample and the probe under a protective acrylic capsule with a thickness of e = 4.40 mm to avoid errors due to airflow or temperature changes for a period of approximately 15 to 25 min per sample until the results were obtained.

**Figure 7:**
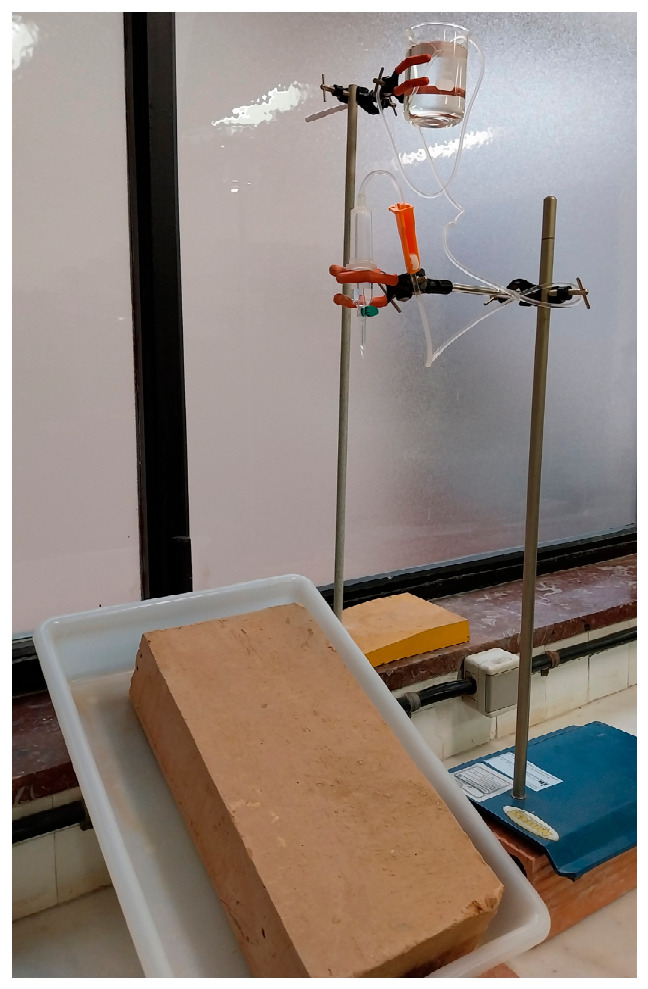
Drip erosion test setup.

The exposure of this type of masonry block to weather requires performing a drip erosion test. Due to the typology of the matrices (non-stabilized constitution, similar to rammed earth), the NZS 4298:2024 [68] standard was applied. Here, 100 mL of water drips from a height of 40 cm, impacting the face of a block positioned with an inclination of 2:1 in a test time of no less than 20 min; six specimens with nominal dimensions of 10 × 20 × 40 cm were tested (Figure 7), noting the average values and the standard error.

**Figure 8:**
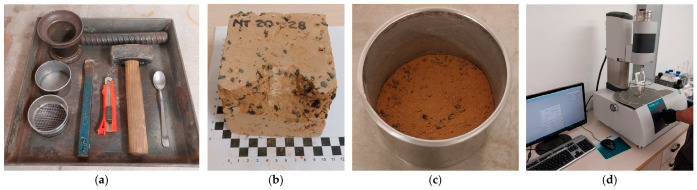
(**a**) Equipment used for sample extraction and crushing. (**b**) Extraction of a representative section of matrices. (**c**) Crushing. (**d**) TG analysis.

A TGA was carried out, a test that enables determining the following in a representative sample of the matrices: composition according to the type of degradation or disintegration, behavior of the compound in the face of increasing temperature, and the amounts of the matrix’s composition as a function of the sample’s weight variation. The test used 5 g samples that were representative of the sizes generated by manually shredding a sample (Figure 8). The temperature range (T) was set from ambient T to T = 1000 °C using a Netzsch thermogravimetric analyzer, model STA 449 F5 Jupiter, from Selb, Germany, with a steady increase in heating rate of 10 °C/min in a hydrogen atmosphere. The choice of a hydrogen atmosphere in the TGA test is based on its ability to generate reducing conditions during the thermal degradation of rubber. Unlike conventionally used inert atmospheres, hydrogen can participate in hydrogenation and hydrogenolysis reactions of the free radicals formed during polymer chain cleavage, modifying the degradation kinetics and the formation of carbonaceous residues. Previous studies on tire hydropyrolysis have shown that the presence of hydrogen significantly alters the thermal decomposition pathways and the distribution of the resulting products [86,87]. The use of hydrogen also allows the evaluation of possible catalytic effects of the minerals present in the soil on the degradation reactions of rubber under reducing conditions, simulating scenarios of advanced thermochemical valorization and hydropyrolysis [86,88].

To correlate the analysis of physical, mechanical, and thermal properties, an optical image analysis was proposed, allowing for the study of matrices based on their physical constitution. Following previous research [61,85,89], the following procedure was used: Samples with approximate dimensions of 2 × 2 × 1 cm were prepared from mid-sections of other untested specimens by cutting with a diamond saw. The samples were embedded in epoxy resin, and after drying and hardening, they were polished with different grit sandpapers (100 to 500) and finally with carbonite polishing compound. The resulting images were produced in JPEG (Joint Photographic Experts Group) format for each matrix under study. These were acquired using an EPSON GT-20000 EU-45 scanner (Suwa, Japan). The optical analysis was performed using Image-Pro v. 11.0.4 Build 9821 software.

In cases where statistical data processing was required, Statistical Software for Social Sciences (SPSS) v. 29.0.0.0 (241) was used, and Solver Software version 2025 Q1 [90] was used for numerical regression adjustment.

## 4. Results

### 4.1. Bulk Density, Apparent Solid Density and Porosity Test Results

**Figure 9:**
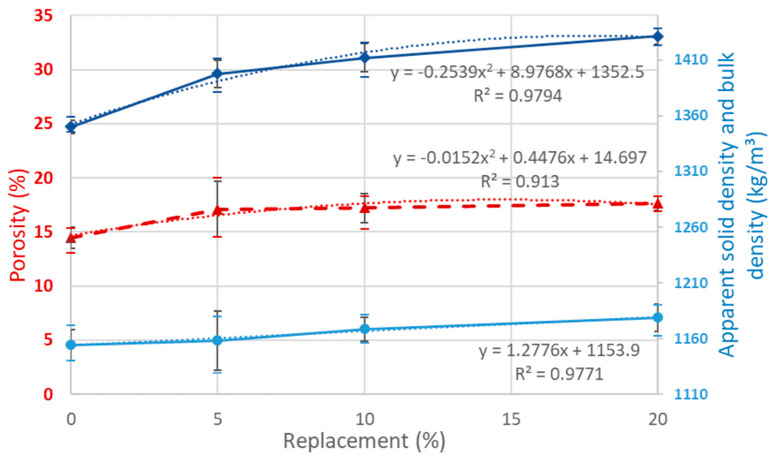
Apparent solid density, bulk density and pore content in samples with CTW as an aggregate replacement. The standard deviation is shown in vertical black lines, and the maximum and minimum values obtained are shown in horizontal lines (with the corresponding colors for each result). The information in EB0 is taken from a previous study [61].

Figure 9 shows the results of the pore content (in red), with values of 14.49, 17.11, 17.23 and 17.63%, respectively, for EB0, EB5, EB10 and EB20. This increase is attributable to the amount of trapped air generated by the new aggregate; although it is not proportional to the replacement percentages, a maximum pore value of 18.33% is reported in EB5. The values recorded for EB10 and EB20 are similar, with low slope between the average values of these matrices. The type of agglomeration or distribution of the new aggregate in the matrix may be the cause; R^2^ = 0.913 shows a good correlation of the average results obtained. It is seen that the values of the present study are more evident than the values of other similar studies (10.85 and 11.75% for 10 and 20% replacement [91]).

The apparent solid density values are 1350.03, 1397.6, 1411.94 and 1431.29 kg/m^3^, respectively, for EB0, EB5, EB10 and EB20, which represent an increase in values of 3.52, 4.59 and 6.01% with respect to EB0. The improved accessibility of the test fluid in the fine pores and interfaces (due to the lower surface tension of the alcohol) is responsible for the progressive increase in this value, despite the lower intrinsic density of the CTW. This, in turn, improves the volumetric characterization of the solid phase, which is determined more by the accessibility of the macro, meso, and micro pores than by the association with the replacement of the lower density aggregate.

The average bulk density values are 1154.44, 1158.20, 1168.53 and 1178.97 kg/m^3^ respectively, which shows an increase of up to 2.12% of EB20 with respect to EB0. Although the tire aggregate has an intrinsic density lower than that of the mineral fraction, the bulk density values show constant values for all replacement percentages (values of the same order of magnitude as the experimental dispersion), indicating the upward trend in the average values (with slope = 1.2776). This is attributable to the compensation between the decrease in the density of the aggregate CTW and the gradual improvement of the packing favored by the deformability of the replaced aggregate itself and the particle size distribution, and consequently it shows no significant variation.

### 4.2. Results of the Hygroscopicity Dimensional Variation Test

This test aims to measure the dimensional variations (specifically, the length) of the matrices with respect to moisture loss or gain (such as shrinkage during drying or under conditions of exposure to ambient humidity), in order to assess the likelihood of cracking, the expansiveness of the E+CTW compound in response to moisture, and the stability of monolithic construction elements.

**Figure 10:**
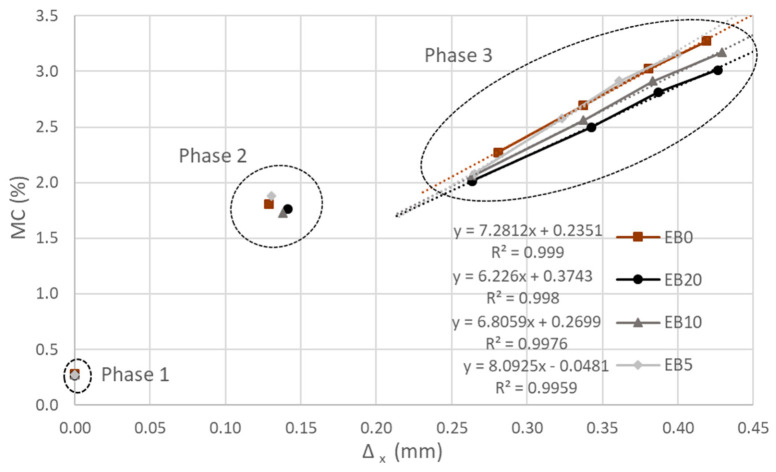
Dimensional variation of the matrices under conditions of ambient humidity saturation. Each phase is shown in dotted circles. The EB0 information is extracted from a previous study [61].

The procedure detailed in previous research [61] was followed, where three phases of dimensional variation are observed (Figure 10). This property is key to understanding the speed and deformation capacity of the new matrices in relation to the moisture absorption capacity.

In the first phase, the matrices are in dry conditions with MC ≈ 0%, after being subjected to kiln drying at 100 °C for 24 h. In the second phase, the values at constant weight and ambient conditions (temperature = 21 °C and RH = 46%) are recorded. Very close average values are observed with reference to EB0 (both in dimensional variation and in MC), values that would correspond to bulk density, demonstrating the stability of this attribute in all matrices under ambient conditions.

The third phase shows the results of exposure to moisture, where the linear regressions demonstrate a high and direct correlation with R^2^ values ≥ 0.99. The slope (s) shows a decreasing trend with a value of s = 6.226. The position of each point of the line of the matrices with CTW with respect to EB0 is below the MC, showing a clear connection between lower MC and higher replacement percentage.

In the case of EB, the clay fraction—which is more sensitive to moisture than the integrated CTW—dominates the phenomenon of shrinkage or dimensional fluctuation, which is mostly caused by drying due to moisture loss.

The incorporation of tire aggregate produced a reduction in the earth blocks’ capacity to absorb moisture. This is mainly due to the partial replacement of the mineral fraction responsible for the volumetric changes during drying as well as the absorption capacity. The tires’ non-absorbent capacity restricts the existing aggregate’s participation in the moisture exchanges, resulting in little interaction between the moisture and the CTW. This can be interpreted as a decrease in the capacity for dimensional variation under the exposure of MC, acting as a buffer against shrinkage under the same conditions and exposure time and lowering the MC to reach similar values to the EB0 range (as shown in Phase 3).

### 4.3. Compressive Strength Test Results

In order to validate the use of CTW as an aggregate in the traditional manufacture of earth blocks, this capacity must be evaluated independently of stabilization or compression. Previous studies have shown a decrease in compressive strength in various types of matrices when incorporating aggregates or tire fibers.

Due to the increased porosity and decreased cohesive capability between the matrix and the new particles of the replaced aggregate, the results exhibit an inverted and declining regressive trend with a higher percentage of replacement of the CTW aggregate.

**Figure 11:**
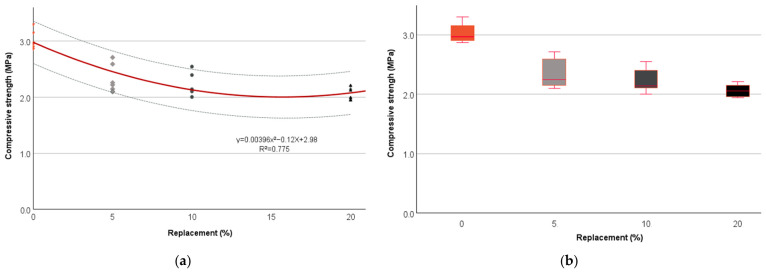
Compression test results. (**a**) *fc* ratios of study matrices. (**b**) Boxplot of compressive strength data. The EB0 information is extracted from a previous study [61].

The hypothetical percentage of replacement in the regression equation, as shown in Figure 11a, is between 10 and 20%, a value where the lower critical point is reached; however, a decreasing trend is observed with a higher percentage of replacement, a phenomenon that should be investigated as a line of complementary research.

The boxplot in Figure 11b displays the median of the results, indicating an abrupt decrease from the 5% replacement. The size of the boxes and whiskers show a decrease in the data dispersion with a higher percentage of replacement, which can be attributed to either the homogeneity of the mixture in the developed matrices or the manufacturing process.

The E-080 [52] and NZ 4298:2024 [68] standards are used as references in traditional production to verify the compressive strength capacity. Of these, the results satisfy the minimum regulatory parameter (1.0 and 0.9 Mpa, respectively). The percentages of compressive strength reduction obtained match the findings of comparable studies in mixtures of stabilized earth and tire aggregates [92,93,94].

It should be noted that the clay of the earth matrix determines the cohesive capacity provided by the compressive strength. Therefore, it is not advisable to use or replace CTW in soils with low cohesive capacity, as the type of clay (with clay and cohesive capacity) used in manufacturing is crucial for achieving results that conform to the regulatory parameters.

### 4.4. Three-Point Flexural Strength Test Results

**Figure 12:**
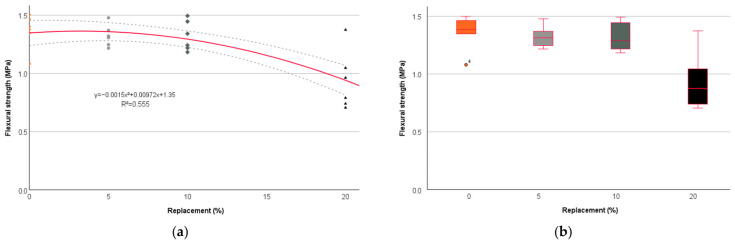
Relationship of *fb* to the study matrices. (**a**) *fb* ratios of study matrices. (**b**) Boxplot of bending strength data. EB0 in accordance with [61].

Figure 12a shows the results of the flexural test, where the results obtained exceeded the minimum limits required by the New Mexico Standard NMAC 14.7.4 (minimum limit of flexural strength) [95]. EB0, EB5 and EB10 have similar and semi-constant average results, with a notable decrease in strength from EB10 to EB20, showing a decreasing trend towards a greater degree of replacement. Although no unstabilized EB investigations are found, there is some agreement regarding the trend and proportion of the decrease in flexural strength of concrete matrices that incorporate granulated rubber [96].

The boxplot in Figure 12b shows the median of the results, which show the abrupt reduction of the flexural strength at a higher percentage of replacement (EB20). The dimensions of the boxes and whiskers show a reduction in the dispersion of the data at a higher percentage of replacement. This is also attributable to the manufacturing process itself and to the homogeneity of the mixture in the developed matrices, consistent with the results of the compression test.

### 4.5. Ultrasonic Elastic Modulus Evaluation Test Results

**Figure 13:**
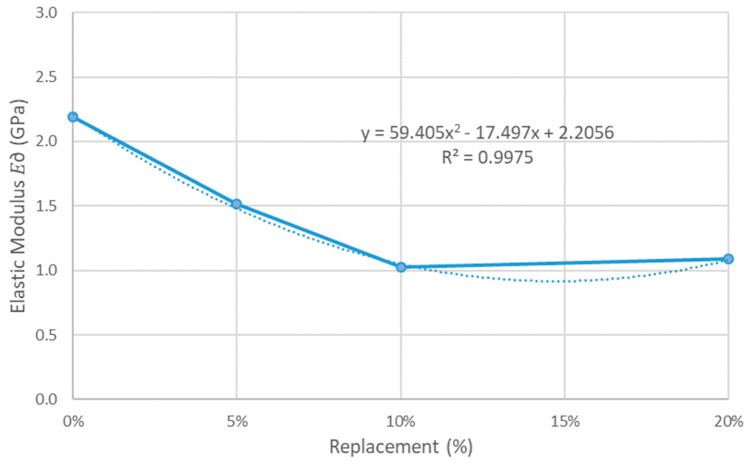
Values obtained from E∂ for the different matrices. Trend in dotted lines. The EB0 information is extracted from a previous study [61].

Due to the similarity to a previous study [61], in order to determine the elastic modulus (E∂), the established methodology to determine the longitudinal strain constant with respect to the transverse constant (μ = Poisson) is applied, by selecting the coefficient identified by Miccoli [97], coupling the values obtained after a statistical analysis. The results are 2.18, 1.51, 1.02 and 1.08 for EB0 (extracted from [61]), EB5, EB10 and EB20, respectively. These results (Figure 13) are consistent with those of the compression test, with the same decreasing trend and a high R^2^ = 0.9975. The initial decrease in the elastic modulus of the soil matrices with partial replacements of CTW can be attributed to the strong contrast in stiffness between the mineral matrix and the considerably more deformable rubber particles. At low replacement levels (5–10%), the incorporation of rubber disrupts the network of rigid soil–soil contacts and generates soil–rubber contacts with lower resistance capacity, causing a significant reduction in the overall stiffness of the material. However, at higher levels (around 20%), the granular system tends to reorganize into a new, more stable hybrid structure, in which stress redistribution, confinement between particles, and partial void filling limit further stiffness loss. Furthermore, the interaction between rubber particles and the confinement effect induce a behavior less sensitive to subsequent increases in the volumetric fraction of shredded tires, leading to a stabilization of the elastic modulus.

### 4.6. Thermal Property Results: Thermal Conductivity, Diffusivity, and Specific Heat

**Figure 14:**
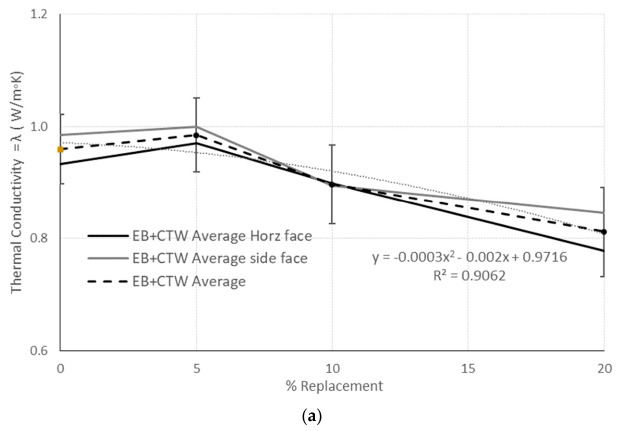

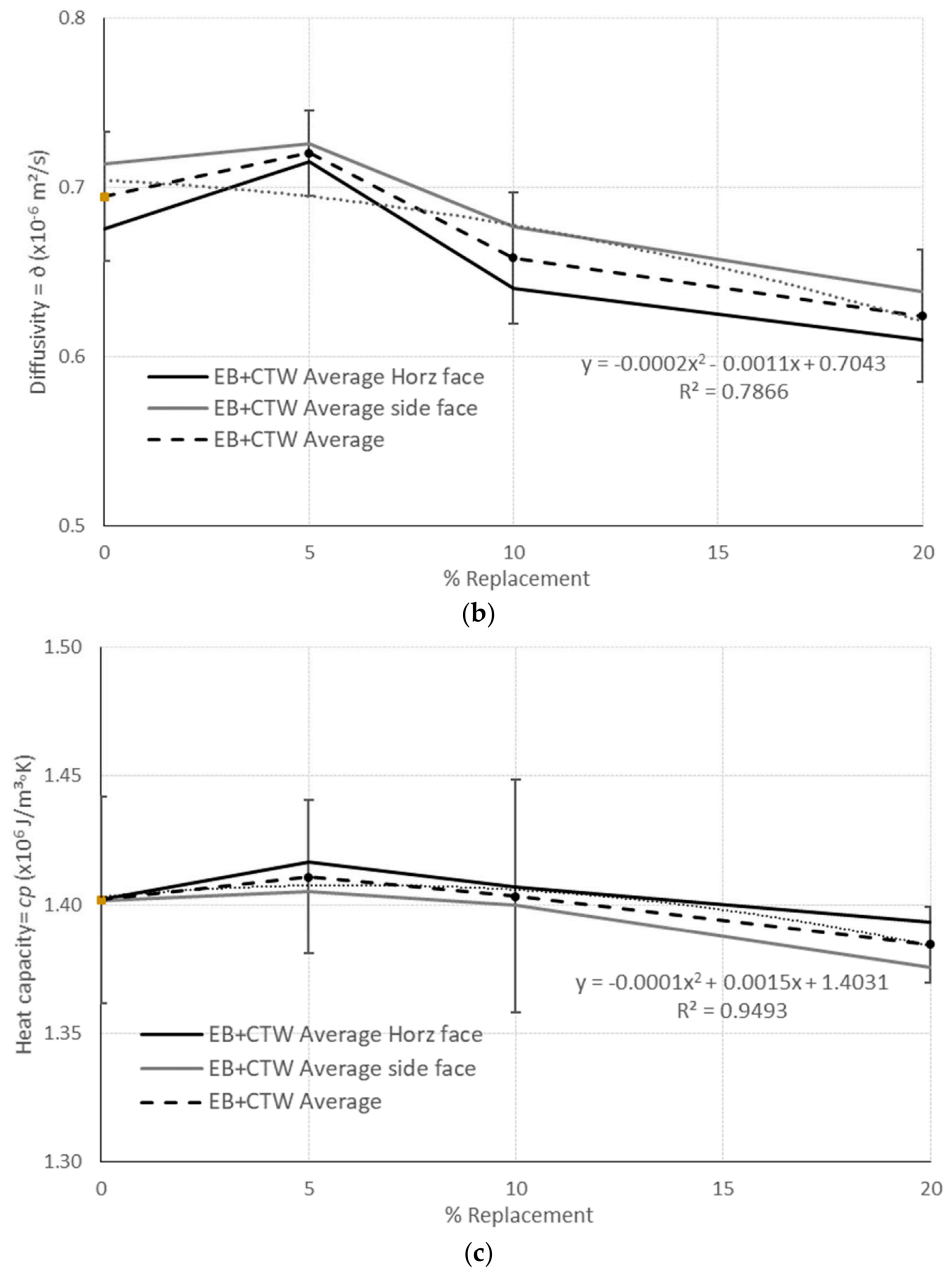
Thermal property test results: (**a**) thermal conductivity (EB0 information is extracted from [85]), (**b**) thermal diffusivity, (**c**) heat capacity. Trends in dotted lines.

The incorporation of the CTW reduces the λ (Figure 14a) of the matrices with earth by an average of 0.812 (W/m°C) in EB20, although the minimum experimental value obtained is reported as 0.66 (W/m°C) for the same replacement percentage; this means a reduction of 15.42% with respect to EB0 and 31.25% of the minimum value obtained. These values are similar to those obtained in earth block research [98,99,100].

The observed behavior of the thermal properties can be explained by the interaction between the material’s microstructure, the void distribution, and the intrinsic properties of the regrind rubber. At low replacement contents (e.g., 5%), the slight initial increase in thermal conductivity may be associated with improved particle packing and partial void filling, which favors the continuity of heat transfer pathways within the matrix. Furthermore, better local compaction can increase the effective contact area between particles, reducing interfacial thermal resistance. However, as the regrind content increases, the effect of rubber as a material with low thermal conductivity and a greater capacity to trap air begins to predominate, generating a progressive interruption of the solid conductive pathways. This results in a gradual decrease in the overall thermal conductivity of the compound.

This phenomenon is indirectly related to the evolution of the elastic modulus, as both parameters depend strongly on the internal microstructure and the connectivity between solid particles. In both cases, the initial substitutions significantly modify the contact network of the granular skeleton. However, while the elastic modulus responds primarily to the mechanical transmission of stresses, the thermal conductivity depends on the continuity of heat transfer pathways. Therefore, although both behaviors are governed by similar microstructural changes —such as granular reorganization, void variation, and the substitution of mineral–mineral contacts with soil–rubber contacts— their trends do not necessarily evolve identically.

The overall decreasing trend in the properties is also attributed to the resulting increase in overall porosity, as well as the number of particles and the low density corresponding to the tire aggregate.

Compared to the results of stabilized blocks from previous research [4] (3% tire aggregate in stabilized blocks), there is a reduction of 43.87% in the average value. These values are due to the difference in the stabilization method (which results in a higher level of porosity) as well as to the replacement percentage.

Similarly, the incorporation of the CTW itself has a greater influence on the thermal diffusivity of the EB with the CTW aggregate (Figure 14b) with the highest degree of replacement percentage, averaging 0.624 × 10^−6^ m^2^/s in the EB20 specimens, or a 10% reduction with respect to the EB0 average.

The values of the resulting caloric capacity (Figure 14c) were also reduced, showing a decrease, with an average minimum value of 1.384 × 10^6^ J/m^3^ × K, which indicates a reduction of 1.28% in the caloric capacity compared with the average of the control blocks. This value is similar to those obtained in previous studies of blocks of compressed raw earth [101].

### 4.7. Drip Erosion Test Results

**Figure 15:**
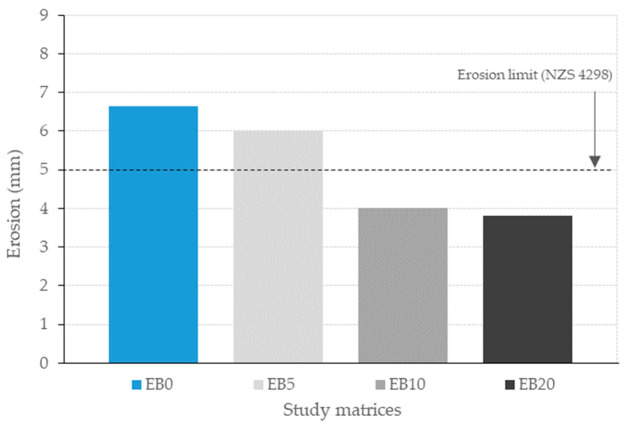
Erosion of the study matrices. The EB0 information is extracted from a previous study [85].

Figure 15 shows the results of the erosion test with the minimum value established by the NZS 4298 standard [68]. The EB0 value is extracted from [85] as a reference value. The results of the matrices incorporating CTW show a decreasing trend inversely proportional to the percentage of aggregate replacement; the higher the CTW, the lower the erosion recorded, which is 9.55, 39.70 and 42.71% lower than EB0 for EB5, EB10 and EB20. In contrast to EB5 (which has the lowest CTW; its behavior is reduced or identical to EB0), the results of EB10 and EB20 exceed the regulatory criteria. The behavior of the matrices with the highest replacement is explained by the presence of CTW particles, where the amount and level of tire aggregate reduce the degree of erosion. This creates a barrier against the soil’s erosion and detachment, acting as a buffer against the impact of droplets, but it does not prevent moisture from penetrating the matrix binder. For earth coverings with a high CTW content that are not subjected to mechanical loads, this could be a substitute or remedy for erosion phenomena.

### 4.8. TGA Results

To better understand the degrading effect of this kind of matrix under heat exposure, thermogravimetric analysis illustrates the decomposition of CTW particles with the earth, based on the change in mass as a function of temperature.

**Figure 16:**
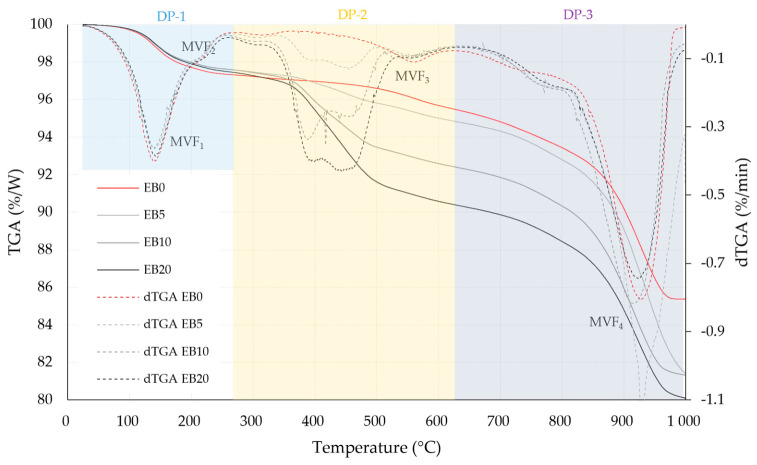
TG and dTGA analysis curves of the matrices incorporating CTW (EB0 is extracted from [61]).

The TGA diagram is shown in Figure 16, where the left vertical axis shows the percentage of mass loss and the right vertical axis shows the derivative of the TGA diagram, using the comparison reference of the TGA curve (control matrix) extracted from [61] and considering the percentage comparison of the areas on the curve obtained by the part integration method.

Phase 1 is characterized by the degradation phase of the earth matrix (described in a prior work [61]) at the start of the test (ambient temperature of 268.75 °C), where the loss of surface water and interlayer water of the minerals themselves is observed. Moo-Young et al. find that tires present stability (with an onset of degradation) at 200 °C [102], although this is not observed in the TGA analysis developed here. Campuzano et al. [103] find that the production of gases such as CO_2_, CO and HO_2_ are also products of decomposition at these temperatures. This result can be attributed to the type of tire, as well as the type of atmosphere used in the test, although [103] the authors find that the production of hydrocarbons generated in an inert atmosphere (N_2_) coincides completely with those obtained under oxidation conditions in the range of 34 to 200 °C.

Phase 2 is defined as a value of 268.75 °C ≤ T ≤ 625.65 °C, a phase in which the degradation of the CTW occurs (evaporation, gasification and decomposition), with volatilization of chemical additives such as plasticizers and processing oils in the tire, where the gases produced from pyrolysis by isomerization/decomposition are CO, CO_2_, NO, SO_2_ and H_2_O [103]. Clearly defined from the beginning of the phase, the literature shows the development of these phenomena from 300 °C [7] to 570 °C, where the greatest loss of mass and the highest peaks in the curves of the derivatives are observed as a consequence of the phenomena associated with the degradation of compounds and types of rubber [7,104,105,106,107]. The percentages of mass losses in this phase correspond to reductions of 8.94, 51.77 and 85.51% with respect to EB0, as the higher replacement rate exacerbates the amount of mass loss in the region within this phase, reflecting accelerated degradation due to the massive volatilization of the polymers. This compromises the structural integrity of the material in these thermal gradients.

In stage 3, there is an increase in the curve of the derivative of 780 °C, which may be due to the thermal decomposition and combustion residues of butadiene-styrene rubber and butadiene rubber [108]; the gases produced in this stage are NO, CO, CO_2_, NO, SO_2_ and H_2_O [103]. The largest peaks of this stage correspond to the endothermic decomposition of the typical earth matrix carbonates, as described in a previous study [61].

The total mass losses as a function of the area on the curves are 10.48, 41.66 and 63.88% higher than EB0. This shows that the total mass loss would be related to thermal degradation as the replacement percentage increases, which is stable up to temperatures close to 250 °C. This is largely determined by the content of the polymeric phase, whose degradation range occurs at higher temperatures than those associated with the removal of water and the dehydroxylation of the mineral matrix.

### 4.9. Microstructural Optical Analysis

An Optical Image Analysis (OIA) of the studied matrices with different CTW contents was carried out to verify the effects of CTW on the EB structure, as well as its predictive capacity on the behavior of some of the properties studied. The guidelines of this study are based on the recommendations of previous works [61,85,89]. Images of the matrices in BitMaP or Bit Mapped Picture (BMP) format were used, obtained using a Seiko Epson GT20000/EU-45 scanner (Suwa, Nagano, Japan). The exposed surfaces of each tablet were previously subjected to metallographic polishing and contained the matrices encapsulated in epoxy resin. Considering the study surface of each tablet, the maximum resolution allowed by the scanner was chosen as 7200 dots per inch (dpi). The images analyzed were in color at 24 bits per pixel, which allows each pixel to represent up to 2^24^ different colors, reaching a theoretical range of approximately 16.7 million colors. The OIA was performed using these images, establishing a procedure in accordance with the objectives set and applying the Image-Pro v. 11.0.4 Build 9821 software.

**Figure 17:**
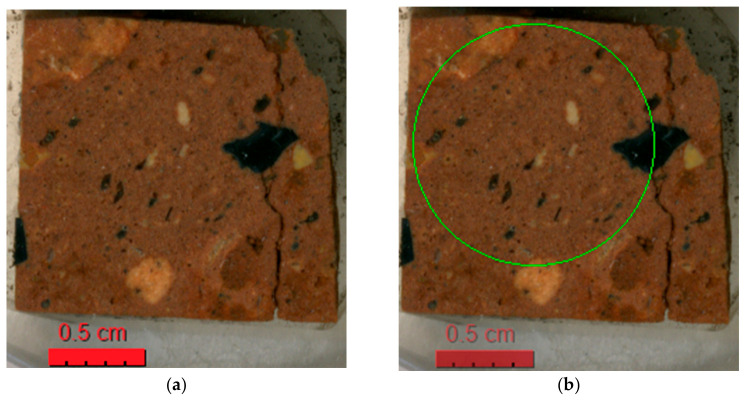
Example of a scanned image of the polished face for the EB5 matrix. (**a**) Scanned image of the polished surface. (**b**) IZ for OIA study (green circle).

To carry out the analysis, an interest zone (IZ) was established in each study matrix, thus delimiting the area to be analyzed. An example is shown in Figure 17, where the IOA is to be applied to the green circle in image b. The IZs were selected in order to avoid the effects of damage caused by the polishing process itself (CTW and E have different stiffnesses, which causes the CTW to deform when subjected to friction, leading to cracks or flaws in the sample’s E). This was more pronounced in the case of EB10, specifically requiring the use of a smaller IZ (43.84% of the average IZ of the different EBs). This meant that the statistical strength of the study was lowered; however, in this case, acceptable results were obtained (see details in the following sections).

**Figure 18:**
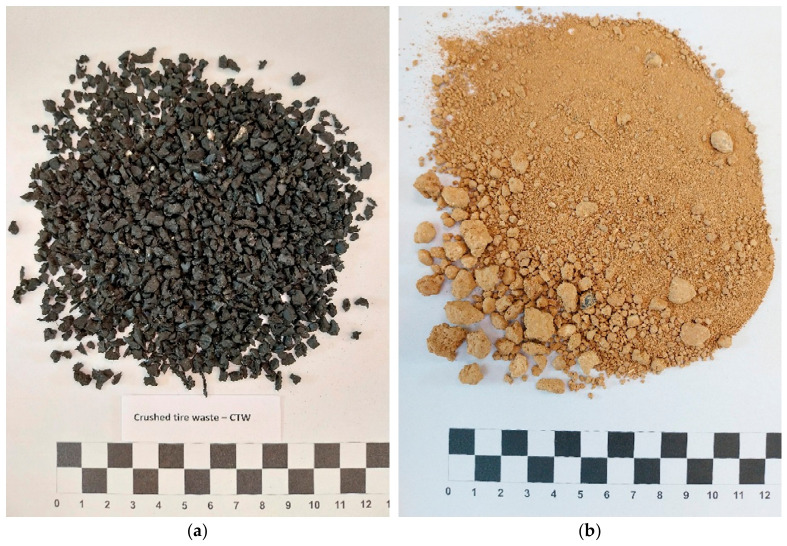
Images: (**a**) CTW and (**b**) E used for OIA calibration.

Photographs of the material components were used in order to calibrate them; Figure 18 shows images of both CTW and E. Similarly, the porosity values of Figure 9 were used to contrast the correct approximation of the OIA process. The initial calibration ranges for the study IZs were established using the histograms of the CTW and E images and considering the criteria of the mono interpretation diagram: the shape and asymmetry of the histogram of intensities and the Maximum Peak Intensity (MPI).

As a result, four different components of the matrices (Cm) were determined:

Cm 1: ‘CTW’ is colored gray for its correct detection or differentiation with porosity ‘black’. This Cm is surrounded by voids (porosity) and is easily identified within the study matrices. The fact that the CTW contour is always surrounded by porosity leads to the formation of Interfacial Transition Zones (ITZs), which are associated with weak areas within the matrix and may cause cracks and rupture due to mechanical stresses. Generally, this component is composed of large particles of a single grain of CTW, which only come into contact with each other when highly concentrated. Its distribution within the IZ is uniform and continuous, with no lumps or agglomerations being identified.

Cm 2: ‘E’ is assigned light brown, as it is the representative sample. This Cm, of all the IZs studied, is considered to be the “support” onto which the other Cms are fitted. It is widely distributed within the IZ, surrounding the rest of the Cms.

Cm 3: ‘white aggregate’ was assigned the color white because it is representative and because it facilitates contrast in the OIA study. This Cm was only identified in the control sample (EB0) and was established in previous research [61]. It was not identified in the rest of the samples (EB5, EB10 and EB20), perhaps because the batches were manufactured at different times. However, its presence within the IZ is reduced or insignificant, not being linked to the behavior of the EBs.

Cm 4: ‘porosity’ was assigned to the IZ spaces in which none of the Cms described above were established; black was used to identify it. This Cm must be assimilated as the absence of solid compounds (Cm 1, Cm 2 or Cm 3), and therefore it is linked to the porosity of the matrix.

**Figure 19:**
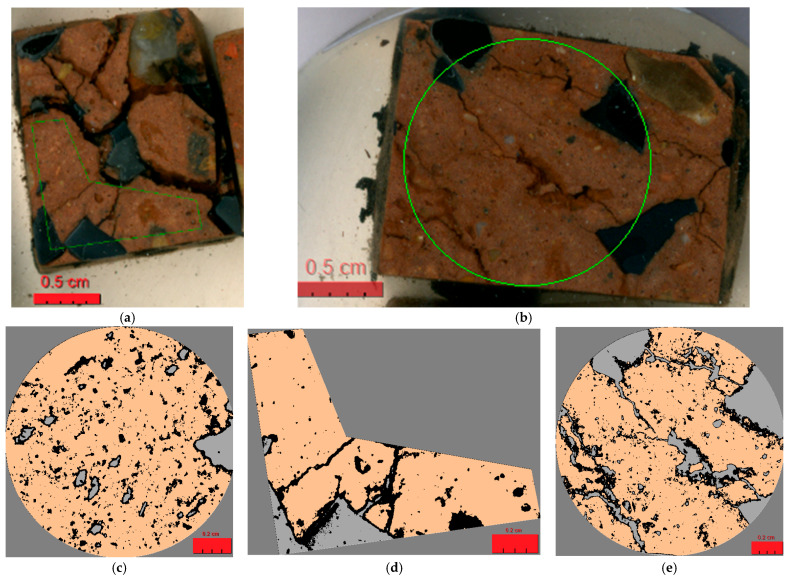
OIA images of the EB. (**a**,**b**) Matrices show the IZs established for the study of BT10 and BT20 (BT5 was previously presented). (**c**) BT5 (IZ = 1.1549 cm^2^). (**d**) BT10 (IZ = 0.592 cm^2^). (**e**) BT20 (IZ = 1.7016 cm^2^). The BT0 matrix was presented in previous work [61].

After establishing the OIA protocols that allow each Cm within the study IZ to be distinguished, their areas were determined in percentage terms and with reference to the total area of the IZ of each sample. The average area of the different IZs studied was 1.1606 cm^2^ (with σ = 0.6172). Figure 19 shows the result of the OIA process of the study EB matrices after the protocols and calibrations had been applied; the figure indicates the areas of each IZ established, reaching an average IZ control of 4.3502 cm^2^ (including that of the EB) [61].

**Figure 20:**
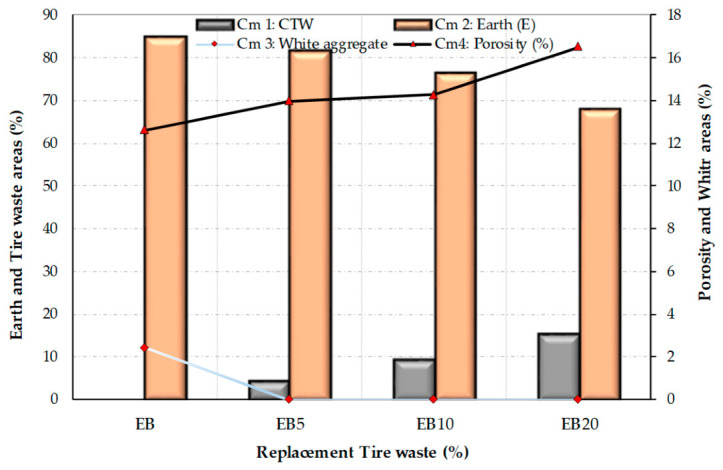
Established percentage areas of the protocol applied in OIA for the IZs of the EB matrices studied.

Figure 20 shows the various percentage Cms calculated in relation to the research EBs with various CTW contents.

From the area percentages obtained for each phase identified in the matrix, we calculated the respective correlation equations along with the coefficients R^2^. Linear regressions were used, considering that the system had three experimental determinations for each variable analyzed. This made it possible to avoid perfect adjustments that could be generated with other regression models, which would hinder establishing clear criteria to differentiate the Cm studied (classified according to [109]).

The correlations obtained, together with their meaning (“+” for directly proportional relation and “−” for inversely proportional), were as follows: for Cm 1, an R^2^ = 0.9938 (strong or very strong correlation) (+); for Cm 2, an R^2^ = 0.9594 (strong and very strong correlation) (−); it was not determined for Cm 3, due to the lack of representation in the IZ of the variables; for Cm 4, an R^2^ = 0.9184 (strong or very strong correlation) (+); and, finally, for Cm 5, an R^2^ = 0.6982 (strong or very strong correlation) (−).

With the above, the following arguments can be presented:

Cm 1 (CTW), the curve that describes the determination of the area percentages in the different study matrices’ IZ has its best fit in an increasing linear equation (the best fit of all the Cms studied: R^2^ = 0.9938). If the values obtained by OIA are compared with respect to the replacement percentages of CTW in their dosage, those of OIA are slightly higher, reaching an average value for all matrices equal to +0.1168% (range of variation between 0.0 and +0.23917%, the latter in the case of EB20). Although the values do not converge strictly in both techniques, the trend of the curve of Cm 1 could best explain the behavior of the properties studied for each of the matrices (inverse relationship). This is based on not only having a directly inverse adjustment that coincides with the loss trends of the established properties but also a percentage of representativeness correlated to them.

Cm 2 Earth (E) is the most representative Cm in the matrices studied; its regression fit is of the decreasing linear type, and it is inversely correlative to the replacement of CTW. This means that this Cm is inversely related to the behavior results of the physical and mechanical properties of the different EBs.

Cm 3 (white aggregate) was identified only in the reference matrix (EB0), with a representative value of 2.42% IZ in the OIA study; therefore, its presence is not significant or representative of the behavior of the EBs.

Finally, Cm 4 (porosity) percentages are established, similar to those indicated in Figure 9; all the results from this Cm 4, obtained by OIA, have been positioned below the experimental ones. Comparing both methods of determining porosity, and considering the average value of all matrices, the OIA is −0.161% (with a range of −0.147 to −0.224%) lower than the experimental results. The observed distribution of porosity is varied due to the different pore sizes, although the smaller sizes predominate. In the case of the EB10 and EB20 matrices, however, porosity is concentrated or grouped into areas with larger pore sizes. This could be explained if the CTW component (with a mostly uniform coarse particle size) was able to “attract” small pores; but Cm 4 does not allow a uniform isotropic matrix (creating a concentration of porous areas and repelling a closer bond with the component Cm 2 (E)) when the CTW concentration is high. This could be explained by showing that the increase in CTW produces an increase in porosity in its matrix, causing it to range from small to large pores.

The prediction equations of these behaviors, and the eigencoefficients that they would have for better calibration of the CTW replacement percentage, were established in order to ascertain the numerical relationship that the various Cms of the EBs have with respect to their previously determined properties. Subsequently, equations were obtained to predict the behavior of the bulk density, fc, and the λ of the EBs studied. The first two properties were chosen because they are the normative limits or acceptance criteria, while the third was chosen as it is the particular goal of this study.

Equations were selected for the linear type adjustment, with the behavior of the EB in each property to be predicted from the conjunction of the four Cms established in OIA. The equation, therefore, presents its components as the sum of each percentage of Cms. The criteria were as follows: (a) each percentage of Cm will be adjusted with a multiplier coefficient, and (b) the Predictive Equation (PE) must in turn be adjusted, when necessary, by a coefficient that considers the CTW contents of each EB studied. Each of the PEs was obtained using the following typology (see Equation (1)):

(1) $$PE={RC}_{CTW}\cdot \left(C_{Cm\;1}\cdot Cm\;1+C_{Cm\;2}\cdot Cm\;2+C_{Cm\;3}\cdot Cm\;3+C_{Cm\;4}\cdot Cm\;4\right)$$

where PE = value of one of the three selected predictive properties of each of the EBs, RC_CTW_ = replacement coefficient of CTW, C_Cm_ i = coefficient determined by numerical adjustment for each of the four Cms as established in the IZ by OIA, and Cm i = percentage of the area of each Cm determined in the IZ by OIA.

Solver software was used to solve the different PEs. Solver version 2025 Q1 [90] has approximation adjustment algorithms with cycle sequencing and optimization correction criteria; the software allows different alternative scenarios capable of establishing the best fit (taking into account the parameters imposed from the start). The C_Cm_ i and the RC_CTW_ corresponding to the studied property of the EBs are established once the parameters imposed for the adjustment have been included and the EP results have been validated. The parameters, the maximum and minimum possible values of each variable according to the experimental values obtained (see Figure 9, Figure 11 and Figure 14a), the approximation criteria determined by the Solver software, the non-linear resolution method, the algorithm iteration parameters used (minimum acceptable convergence error value of 0.0001), the evaluation of I + 1 iteration by approximation (in forward difference) and the initial test value of the variables to be evaluated (all equal to zero) followed the recommendations of previous research [110].

Once the EPs were obtained, they were contrasted with the experimental results, thus determining the average error of the predictive accuracy (EPA), which allowed the predictions with the best approximation to the real experimental results to be chosen. The following PEs include the coefficients Cm i that were established: for bulk density, see Equation 2 (EPA = 0.324 kg/m^3^), for *fc* see Equation (3) (EPA = −0.0012 MPa) and for *λ* see Equation 5 (EPA = −0.0007 W/m·K):

(2) $$Bulk\;Density={RC}_{CTW}\;\left(16.59\cdot C_{m}\;1+13.72\cdot C_{m}\;2-0.36\cdot C_{m}3\cdot \;14.65\cdot C_{m}\;4\right)$$

(3) $$F_{ck}={RC}_{CTW}\cdot \left(0.0335\cdot CM\;1+0.052\cdot CM\;2+0.05327\cdot CM\;3+0.029\cdot CM\;4\right)$$

(4) $$\lambda ={RC}_{CTW}\cdot \left(-0.0097\cdot CM\;1+0.00894\cdot CM\;2-0.0276\cdot CM\;3+0.0212\cdot CM\;4\right)$$

Table 1 shows the RC_CTW_ to be used in the above equations for the different EB matrices studied. It should be noted that for both bulk density and λ the established PEs achieve a perfect fit without the use of this coefficient (RC_CTW_ = 1.0), so they can be omitted when the PEs of these properties are used.

Table 2 shows the values of the real Experimental (Ex.) and Predictive (Pr.) properties determined with the PE adjustment and considering the RC_CTW_ for the different EB matrices studied.

**Figure 21:**
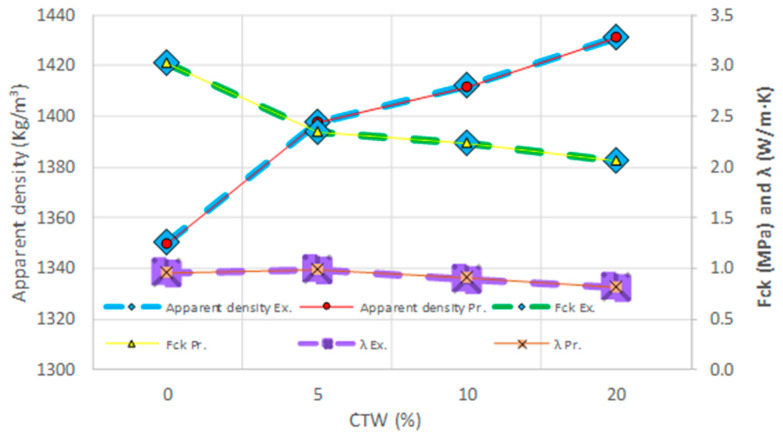
Results of the comparison between the experimental and predictive results of the EB properties for the study matrices that consider CTW.

The experimental and predictive results are graphed in Figure 21 in order to present the degree of fit or variation between them, showing the predictions juxtaposed with the real experimental values.

A sensitivity study of each variable was carried out separately in the determined PEs in order to better understand the regressions, from which the following can be deduced:

1. For bulk density: Cm 1 (CTW) is the dominant variable with the greatest impact (36.6% relative importance (RI)) in determining this PE (with a clear increasing linear relationship), and Cm 3 (white aggregate) has the least impact (without having a clear correlation pattern, 0.8% RI). Cm 2 (E) has an inverse relationship, but with a positive—cross-correlation—coefficient (30.3% RI), while Cm 4 (porosity) has a strong correlation with a positive relationship (32.3% RI). Therefore, it can be deduced that the dominant variables are Cm 1, Cm 4 and Cm 2, and Cm 3 is irrelevant. In addition, the most important variable with the greatest power of PE change is Cm 1 (CTW). The behavior of the variables could be expressed as follows: Cm 1 > Cm 4 > Cm 2 >>> Cm 3. It is concluded that the prediction is linear, predictable and sensitive.
2. For compressive strength (*fC)*: the result of the PE of this property is a linear combination of variables, ranked by the RC_CTW_ coefficient, which acts as a global degradation factor. In this PE the most important —but doubtful—variable was Cm 3 (RI = 60–70% strong and positive but biased); the Cm 2 variable (RI = 20–25% strong positive correlation, high impact) is influential, followed by the Cm 1 (RI = 5–10% apparent negative, with low impact) and Cm 4 variables (RI = 5–10% slight negative, with low impact). It is concluded that the model has a perfect, but over-adjusted, fit; the RC_CTW_ coefficient is a critical coefficient (global control), and Cm 2 is the most reliable variable. The variables could be expressed as follows: Cm 3 (unreliable) > Cm 2 > Cm 1 > Cm 4. It is concluded that the prediction is relatively stable.
3. Finally, for λ: in the resulting PE, the determined coefficients absorb the effect of the RC_CTW_ coefficient. The effects of the variables indicate that Cm 1 has a negative effect with medium impact (RI = 14.4%), Cm 2 has a positive effect with medium-low impact (RI = 13.3%), Cm 3 has a strong —but unreliable—negative effect (RI = 40.9%), and Cm 4 has a positive effect with a high impact (RI = 31.4%). It can be concluded from the above that the model is directional, but not strongly predictive, and the most important and reliable variable is Cm 4. The variables could be expressed as follows: Cm 3 (not stable) > Cm 4 (the most reliable) > Cm 1 > Cm 2.

In order to establish the robustness and applicability of the established PE equations, a statistical study was conducted that included different metrics. The statistical values were calculated using IBM SPSS Statistics software, version: 29.0.0 (241):

1. Nature of the model: A stepwise model was established with the following form:

(5) $$R=\beta_{1}V_{1}+\beta_{2}V_{2}+\beta_{3}V_{3}+\beta_{4}V_{4}$$

where:

(6) $$\beta_{i}V_{i}=CA_{i}$$

2. Model fit evolution:

2.1. Coefficient of determination R^2^ was studied using the following equation and rank classification considerations:


(7) $$R^{2}=1-\frac{\sum {\left(R\exp-Rpred\right)}^{2}}{\sum {\left(R\exp-Rexp\right)}^{2}}$$

R^2^ > 0.9: Excellent.

0.7 < R^2^ < 0.9: Acceptable.

R^2^ < 0.7: Weak model.

A detailed analysis of R^2^ is presented later.

2.2. Root Mean Square Error (RMSE):

For this case, the average error of the model was evaluated using the following equation:

(8) $$RMSE=\;\sqrt{\frac{1}{n}\sum {\left(R\exp-Rpred\right)}^{2}}$$

The key consideration with this metric is that the lower the RMSE, the better the fit.

In this study, the RMSE values were as follows: apparent density = 0.6474, F_ck_ = 0.0025, and λ = 0.0014. Therefore, it can be inferred that this metric performs worst for apparent density, while it performs significantly better for F_ck_ and λ.

3. Residual Analysis:

To determine this metric, the following equation is used:

(9) $$e_{i}=\;R\exp-Rpred$$

In this case, this metric should analyze the following:

3.1. Random distribution: The residuals must meet the following criteria: have a mean close to 0, show no patterns, and be randomly distributed. If trends appear, then the model should be considered incomplete, nonlinear terms may be missing, or interactions between variables may exist. In this research, the average e_i_ values were, for bulk density: 0.3237; for F_ck_: −0.0012; and for λ = 0.0007. Therefore, the distribution is considered random, with an acceptable mean e_i_ and no trends.

3.2. Homoscedasticity: To determine homoscedasticity, the variance (of a sample; σ^2^) of the errors was used, considering that it should remain approximately constant. In this research, σ^2^ was 0.0016 for bulk density, while for F_ck_ and λ it was 0.000. The above can be considered constant, and therefore the model can be considered robust. However, if the error increases with the R^2^ value, the model should be considered to lose robustness.

3.3. Normality of residuals: The Shapiro–Wilk formula (*n* < 50) was used for this assessment. The normality of the residuals yielded the following significance values (*p* < α = 0.05): 0.412 for bulk density, 0.885 for F_ck_, and 0.852 for λ. Therefore, it was assumed that the distribution of the residuals follows a normal distribution.

4. Robustness of the model: To determine this, a perturbation analysis was performed, which introduces experimental noise using the following equation:

(10) $$Vi^{\prime }=V_{i}\left(1+\epsilon \right)$$

The values of ϵ are usually set at ϵ = 1%, ±5%, and ±10%, and this metric is used to assess the stability of R^2^. If small variations generate large changes in R^2^, the model should be considered fragile. In this research, disturbances of ϵ = ±10% and ±15% were used (because the initial model equations required an error of <5% for acceptance). In this research, the summary R^2^ values for ϵ = ±10% and ±15% were as follows (see Table 3):

Where

R^2^ > 0.9: excellent;

0.7 < R^2^ < 0.9: acceptable;

R^2^ < 0.7: weak model.

As can be seen, the R^2^ coefficients have small variations and, in any case, do not alter the classification criteria of the initial projected fit. It is observed that for the case of apparent density, its coefficient is excellent.

5. Applicability of the model:

This section defines the domain where the model is reliable.

5.1. Valid experimental range: The model is only valid within the intervals used in the fitting:


(11) $$v_{im_{i}n}\leq v_{i}\leq v_{imax}$$

In this investigation, the valid experimental ranges were as follows:

Vi min. (ϵ = −15% Vi) ≤ Vi ≤ Vi max. (ϵ = −15% Vi).

This allows us to consider the predictive equations acceptable within a range of 30% of the experimental values of the study.

5.2. Coverage of experimental space: It must be verified whether all relevant combinations of variables were sampled and whether there are areas without data. In this investigation, the variables and possible combinations were established at the beginning of the investigation, being appropriate for the objective of the investigation and covering usual and admissible replacement values.

5.3. Multicollinearity: It must be verified whether the variables are highly correlated (the coefficients lose physical meaning; the model becomes unstable). To investigate this aspect, correlation matrices or the VIF (Variance Inflation Factor) are usually evaluated. Therefore, a bivariate correlation matrix is proposed with the studied variables; the results of the analysis are expressed below.

Apparent density Ex. vs. F_ck_ Ex. A correlation of −0.991 is obtained.

Apparent density Ex. vs. λ Ex. A correlation of −0.730 is obtained.

F_ck_ Ex. vs. λ Ex. A correlation of 0.630 is obtained.

The results indicate a strong inverse correlation between apparent density and F_ck_. This is noteworthy since the opposite is usually the case in the mechanical behavior of material matrices. In any case, for aspects of multi-collinearity, it seems that this factor must be taken into account, thus reducing the physical meaning of the proposed coefficients.

In summary, the practical indicators of model robustness were as follows:

- High R^2^ values. In this research, all models are classified as excellent or acceptable.
- Low RMSE values. In this study, with the exception of bulk density, the remaining variables obtained very low values.
- Random residuals. In this study, the residual distribution is random, with an acceptable mean and no trends. Regarding homoscedasticity, the σ^2^ values can be considered constant, and therefore the model can be considered robust. The distribution of the variables was determined to be normal.
- Model robustness. The studied disturbances of ε = ±10% and ±15% indicate that the R^2^ coefficients have small variations and, in any case, do not alter the classification criterion of the initial projected fit. It is observed that the coefficient for apparent density is excellent, while for F_ck_ and λ it is acceptable.

Regarding the model’s applicability, the verified metrics were as follows:

- Valid experimental range. The study establishes that the validity of the proposed equations is as follows: Vi min. (ϵ = −15% Vi) ≤ Vi ≤ Vi max. (ϵ = −15% Vi). This allows the predictive equations to be considered acceptable within a range of 30% of the experimental values in the study.
- Coverage of the experimental space. In this research, the variables and possible combinations were established at the beginning of the investigation, being appropriate for the research objective and covering usual and admissible replacement values.
- Multicollinearity. The results of the bivariate matrix analysis establish that there is a strong inverse correlation between apparent density and Fck. This is noteworthy because the opposite is usually the case in the mechanical behavior of material matrices; in any case, for multicollinearity aspects, this factor should be taken into account, thus reducing the physical significance of the proposed coefficients.

## 5. Conclusions

Based on the study of the granulated tire particles used as aggregate in making earth blocks, according to a traditional manufacturing process, the following conclusions were reached.

Previous research demonstrated the usefulness and feasibility of this aggregate in the manufacture of different construction materials, some of which focused on correcting the reduction of resistance by using stabilization methods to achieve resistances according to the regulatory limits. It is observed that the higher pore content and density reduction not only is obtained at higher replacement levels, without compaction, but is also dependent on larger grain size, although with contents of 10 to 20%, this no longer presents major changes.

The incorporation of recycled tire aggregate affects the shrinkage capacity, showing that the rubber particles have a lower moisture retention capacity in the matrices; however, this is reduced by the consequent generation of pores, achieving similar retractions at lower moisture content.

One of the determining properties is the compressive strength, which would help to validate its bearing capacity and use in masonry elements. The results are in accordance with those of previous studies (greater reduction of resistance to higher content of tire particles), which state that its use in load-bearing or structural elements is not recommended. However, this study shows that this capacity is related to the variables in the amount of recycled aggregate used, as well as the cohesive capacity of the earth matrix itself (being recommended in soils with a higher capacity); both achieve results above the normative minimum established for unstabilized earth blocks, considering the applicable regulations explained in the introduction section.

Other authors report superior results when using tire fibers for flexural strength; in this study, however, the use of granulated material has a minimal effect on low replacement contents and rapid decline at high contents.

The most important results achieved are those of the thermal properties, which are reduced at a greater degree of replacement (up to 0.66 (W/m °C) at 20%). This confirms the insulation capacity, being comparable to similar research that incorporates vegetable fibers or other types of waste. This makes it ideal for coating and facade compounds that require reduced thermal behavior, contributing to the erosion resistance values obtained. However, in the case of adobes that require the mass itself for greater thermal inertia, the reductions in conductivity and specific heat would reduce this behavior.

Furthermore, it was found that incorporating shredded tires into soil matrices generates gaseous emissions associated with degradation from heat exposure and oxidation, especially due to the presence of rubber, including CO, CO_2_, NO, and SO_2_. Although a hydrogen atmosphere allows for the study of the thermochemical behavior of soil–tire mixtures under reducing conditions and the exploration of potential interactions between degradation products and hydrogen, the results obtained do not directly represent the service conditions of the construction materials. Furthermore, the chemical participation of hydrogen and the potential catalytic effects of the minerals present in the soil matrix attribute the observed phenomena to the thermal degradation of the composite rather than of the individual components. Therefore, the characteristic temperatures and kinetic parameters determined should be interpreted as specific values for these parameters as well as for the test atmosphere. Further studies are needed on the effect of packing or encapsulation on more soil matrices. Therefore, fire exposure, ignition, and flammability tests, as well as quantitative gas emission analyses, are required. Likewise, a more in-depth study of the prolonged exposure of the coatings to heat and water under outdoor conditions, durability, aging, and post-exposure mechanical properties is necessary. Similarly, microscopic tests are recommended to determine the influence of the new porous network on gas transfer phenomena and degradation kinetics, which opens new lines of research.

Therefore, it can be said that earth blocks with CTW are not recommended for structural uses (such as load-bearing walls) or in elements exposed to fire or temperatures, being ideal for applications where they can be used as thermal insulation or in partitions without considerable loads, reiterating the need for further studies for safe purposes in construction. To conclude with the image analysis, it is established that the OIA technique is considered complementary, with the ability to identify the components of a microstructure of matrices like those studied. However, it is also possible to determine correlations and predictions of the physical, mechanical or thermal behavior of the matrices by numerically adjusting equations, obtaining information from this technique with the initial data of the numerical adjustments.

In conclusion, the analysis of the robustness and applicability of the predictive equations proposed in the research indicates that the proposed equations for making predictions are robust. With respect to their applicability, the range is sufficiently broad for the possible variations of the variables, covers common variables and combinations of these study variables, and takes into account multicollinearity.

## Figures and Tables

**Table 1:** RC_CTW_ adjusted for each EB property considering CTW.

	RC_CTW_ Coefficient for EB Properties		
EB Matrix	Bulk Density	F_ck_	λ
EB0	1.0	0.49820	1.0
EB5	1.0	0.48722	1.0
EB10	1.0	0.47624	1.0
EB20	1.0	0.45428	1.0

**Table 2:** Values of the real experimental (Ex) and predictive (Pr.) properties.

Bulk Density (kg/m^3^)		Compressive Strength (MPa)		Thermal Conductivity (λ W/m·K)	
Bulk Density Ex.	Bulk Density Pr.	*f*c Ex.	*f*c Pr.	λ Ex.	λ Pr.
1350.03	1349.67	3.024	3.0255	0.960	0.9604
1397.60	1397.26	2.337	2.3384	0.985	0.9858
1411.94	1411.62	2.238	2.2392	0.897	0.8981
1431.29	1431.03	2.061	2.0619	0.812	0.8127

**Table 3:** Robustness study of the model by perturbation of the variables.

R^2^ for Apparent Density			R^2^ for F_ck_			R^2^ for λ		
ϵ = +10%	Projected	ϵ = −10%	ϵ = +10%	Projected	ϵ = −10%	ϵ = +10%	Projected	ϵ = −10%
0.9247	0.9247	0.9247	0.87664	0.8368	0.8368	0.8443	0.7904	0.7114
ϵ = +15%	Projected	ϵ = −15%	ϵ = +15%	Projected	ϵ = −15%	ϵ = +15%	Projected	ϵ = −15%
0.9247	0.9247	0.9247	0.8595	0.8368	0.83689	0.7904	0.7904	0.7904

## Data Availability

The original contributions presented in this study are included in the article. Further inquiries can be directed to the corresponding author.

## Abbreviations

The following abbreviations are used in this manuscript:

BMP	Bit mapped picture
CTW	Crushed tire waste
dpi	dots per inch
E	Earth
EB	Earth block
ELT	End of life tire
EPA	Error of predictive accuracy
Ex	Experimental properties
*fb*	Bending strength
*fc*	Compressive strength
FM	Fineness modulus
FRP	Fibrous rubber particles
GTR	Granulated tire rubber
ITZ	Interfacial transition zone
IZ	Interest zone
LL	Liquid limit
MC	Moisture content
MPI	Maximum peak intensity
NR	Natural rubber
OIA	Optical image analysis
PE	Predictive equation
RA	Recycled aggregate
RC	Replacement coefficient
RH	Relative humidity
RI	Relative importance
RM	Rubber mulch
SBR	Styrene-butadiene copolymer
SEBs	Stabilized earth blocks
T	Temperature
λ	Thermal conductivity
TGA	Thermogravimetric analysis
UPVs	Ultrasonic pulse velocity by wave transmission
